# Engineering of Magnetic Softness and Domain Wall Dynamics of Fe-rich Amorphous Microwires by Stress- induced Magnetic Anisotropy

**DOI:** 10.1038/s41598-019-48755-4

**Published:** 2019-08-27

**Authors:** P. Corte-León, J. M. Blanco, V. Zhukova, M. Ipatov, J. Gonzalez, M. Churyukanova, S. Taskaev, A. Zhukov

**Affiliations:** 1 Dpto. Física de Materiales, Fac. Químicas, UPV/EHU, 20018 San Sebastian, Spain; 2 Dpto. de Física Aplicada, EIG, UPV/EHU, 20018 San Sebastian, Spain; 3 National University of Science and Technology «MISIS», Moscow, 119049 Russia; 4 NRU South Ural State University, Chelyabinsk, 454080 Russia; 5 IKERBASQUE, Basque Foundation for Science, 48011 Bilbao, Spain

**Keywords:** Magnetic properties and materials, Sensors and biosensors

## Abstract

We observed a remarkable improvement of domain wall (DW) mobility, DW velocity, giant magnetoimpedance (GMI) effect and magnetic softening at appropriate stress-annealing conditions. Beneficial effect of stress-annealing on GMI effect and DW dynamics is associated with the induced transverse magnetic anisotropy. An improvement of the circumferential permeability in the nearly surface area of metallic nucleus is evidenced from observed magnetic softening and remarkable GMI effect rising. We assumed that the outer domain shell with transverse magnetic anisotropy associated to stress-annealing induced transverse magnetic anisotropy affects the travelling DW in a similar way as application of transversal bias magnetic field allowing enhancement the DW velocity. Observed decreasing of the half-width of the EMF peak in stress-annealed microwires can be associated to the decreasing of the characteristic DW width. Consequently, stress annealing enabled us to design the magnetic anisotropy distribution beneficial for optimization of either GMI effect or DW dynamics.

## Introduction

Magnetic nano-micro wires can present fast domain wall (DW) propagation observed in diverse families of magnetic wires^1–4^, as well as extremely excellent magnetic softness and giant magnetoimpedance (GMI) effect observed mostly in amorphous and nanocrystalline magnetic microwires^5–8^. These properties of magnetic wires are essentially relevant for emerging industries, i.e., magnetic sensors, electrical engineering, medicine, informatics, magnetic recording, electronic surveillance among others.

To a great extend these properties are related to cylindrical geometry and therefore can be observed in either amorphous or crystalline wires with rather different dimensions^1–5,7–12^. However, amorphous wires prepared using melt quenching provide a number of great advantages, such as excellent magnetic softness combined with better mechanical properties^13,14^.

Indeed, defects of the crystalline structure such as grain boundaries, texture, dislocation density, etc., notably alter the magnetic softness of crystalline materials. Accordingly, optimization of magnetic softness of crystalline magnetic materials involves durable and costly annealing^12,14^. However, magnetic softness of amorphous magnetic materials is originated by the liquid-like structure characterized by the absence of crystalline structure and hence defects typical for crystals^14–16^. Accordingly, excellent magnetic softness can be achieved in as-prepared samples without need of thermal treatments and post-processing. Furthermore, rapid quenching from the melt preparation technique is quite fast and inexpensive^3–5^.

Among other aspects significant for technological applications of soft magnets are the dimensionality, the cost efficiency and the tuneability of magnetic properties. Use of low dimensional soft magnets allows reduction of magnetic devices. For these reason, low dimensional and cost effective soft magnets are especially demanded for a number of emerging applications^8,17–20^. The thinnest amorphous magnetic wires can be prepared by the Taylor Ulitovsky method described elsewhere^8,12,21^. The Taylor Ulitovsky technique involves simultaneous rapid solidification of the metallic nucleus with diameters 0.5–90 μm surrounding by the glass coating with thickness 0.5–20 μm^12,22^.

In spite of the absence of magnetocrystalline anisotropy the other sources of magnetic anisotropy, like magnetoelastic and shape anisotropies determine the magnetic properties of the glass-coated microwires with amorphous structure.

The magnetoelastic anisotropy, *K*_*me*_, is determined by the magnetostriction coefficient, *λ*_*s*_, and the internal stress, *σ*_*i*_, through the following expression^8,14,23^:

1 $${K}_{me}\approx 3/2\,{\lambda }_{s}{\sigma }_{i},$$

The magnetostriction coefficient of as-prepared amorphous magnetic materials is determined mostly by chemical composition of the alloy^24^. High and positive magnetostriction coefficient values are observed in Fe-rich compositions (typically *λ*_*s*_ ≈ 35–40 × 10^−6^). Substitution of Fe by Co or Ni in Co_x_Fe_1−x_ (0 ≤ x ≤ 1) or Ni_x_Fe_1−x_ alloys allows *λ*_*s*_ –values decreasing^23–25^. Amorphous Co-rich alloys present negative λ_*s*_ –values (typically *λ*_*s*_ ≈ −5 × 10^−6^). Therefore, vanishing *λ*_*s*_ –values can be obtained in the Co_x_Fe_1−x_ (0 ≤ x ≤ 1) alloys at x about 0,03–0,08^23–25^. Similarly, in Ni_x_Fe_1−x_ alloys a decrease of *λ*_s_ -values with an increase of the content of Ni is reported.

The other source of magnetoelastic anisotropy - internal stresses is originated by the rapid melt quenching of the metallic alloy ingot itself in addition to the different thermal expansion coefficients of the metallic alloy and glass coating^21,22,26–28^. The internal stresses arising during the glass-coated microwires preparation have been evaluated theoretically and experimentally elsewhere^21,22,26–28^.

All the simulations confirmed by the experimental studies (gradual glass-coating removal by the chemical etching or stress relaxation by annealing) evidenced that the axial stresses are predominant within main part of the metallic core^8,26–28^.

Moreover, the internal stress values decrease with increase in the ratio, *ρ*, between the metallic nucleus diameter, *d*, and the total microwire diameter, *D* (*ρ* = *d/D*)^26–29^.

These factors determine the magnetization distribution and hence domain structure of glass-coated microwires: microwires with positive magnetostriction coefficient present axial easy magnetization direction, while microwires with negative magnetostriction coefficient present circumferential easy magnetization direction^8,28^.

Considering experimental results on dependence of remanent magnetization versus *ρ* –ratio^29^ and magneto-optical studies^30^ it is commonly assumed that the domain structure of glass-coated microwires with positive magnetostriction coefficient consists of an axially magnetized single domain surrounded by the outer domain shell with radial magnetization^29–37^.

Consequently, the magnetic microwires with positive magnetostriction coefficient present the magnetic bistability related to the presence of a large and single axially magnetized inner core. Under application of axial reversal magnetic field the Barkhausen jump takes place. The magnetization reversal runs through quite fast magnetization switching within the single axially magnetized domain by fast DW propagation along the wire^4,31,32^.

However, the domain structure of microwires with negative magnetostriction coefficient consists mainly of circular 180° domains. The inner axially magnetized core volume is rather small but can increase after chemical etching or after annealing allowing relaxation of internal stresses (mainly of tensile character)^8,33^.

The remagnetization process of microwires with negative magnetostriction is therefore associated to the magnetization rotation^12,33^.

Unusually fast (above 1 km/s) DW propagation observed in amorphous and nanocrystalline microwires becomes a topic of intensive research^4,31,32,34–37^. The main interest is related to proposed applications of fast and controllable domain wall propagation in magnetic recording, electronic surveillance and magnetic sensors^1,2^. For such applications, the DW works as the memory element or logic gate. Naturally the speed of DW propagation and control of DW movement are essentially relevant for proposed applications.

The origins of extremely high DW velocities in amorphous and nanocrystalline microwires are discussed elsewhere in terms of perfectly cylindrical shape, absence of magnetocrystalline anisotropy and peculiar domain structure of glass-coated microwires^4,31–37^. In particular the DW velocity can be remarkably improved by decreasing the magnetoelastic anisotropy either through the selection of metallic nucleus composition with lower *λ*_*s*_ –values or by internal stresses relaxation through the appropriate annealing^26,31,33,38^.

However, the propagating DW in magnetic microwires is essentially not abrupt: recent studies reveal that the DWs have a complex structure with rather large width determined by the magnetoelastic anisotropy and metallic nucleus diameter^39–41^. In particular, an increasing of the characteristic width of a head-to-head DW with decreasing of the anisotropy constant, *K*, is reported^39^. Either conical or planar DW shape is considered^39,41^.

The micromagnetic origin of the DW in magnetic microwires is still unclear. However, in spite of extremely fast DW propagation observed in low magnetostrictive microwires such extended DWs are unsuitable for aforementioned applications.

Therefore, the other possibilities allowing enhancement of the DW velocity are exploiting. One of the possibilities related to the internal stresses relaxation in highly magnetostrictive Fe-rich microwires allowed considerable DW velocity and mobility enhancement^31,33^. Alternatively, theoretically and experimentally was shown that the DW velocity in a variety of groups of magnetic microwires can be considerably improved under application of transversal bias magnetic field^36,37,42,43^. Effect of transversal magnetic field on DW velocity is discussed considering the influence of transverse magnetic anisotropy on DW dynamics, influence of transverse field on spin precession and change of the DW width^42,43^.

Recently, the onset of the transverse magnetic anisotropy in highly magnetostrictive Fe-rich magnetic microwires is reported^44,45^. In particular, at certain stress-annealing conditions the axial magnetic anisotropy can be still maintained in main part of the metallic nucleus, while the outer domain shell with transversal magnetic anisotropy can be created^44,46^. Such transversal magnetic anisotropy is evidenced by change of hysteresis loops and drastic increasing of magnetoimpedance effect^44–46^.

From the viewpoint of fast DW propagation observed in microwires looks promising, but up to now DW dynamics in such microwires with stress-induced transverse magnetic anisotropy was not studied.

Consequently, in this paper we present new experimental results on influence of stress-induced anisotropy on domain wall dynamics, GMI effect and magnetic softness of Fe- based glass-coated microwires.

## Results and Discussion

**Figure 1:**
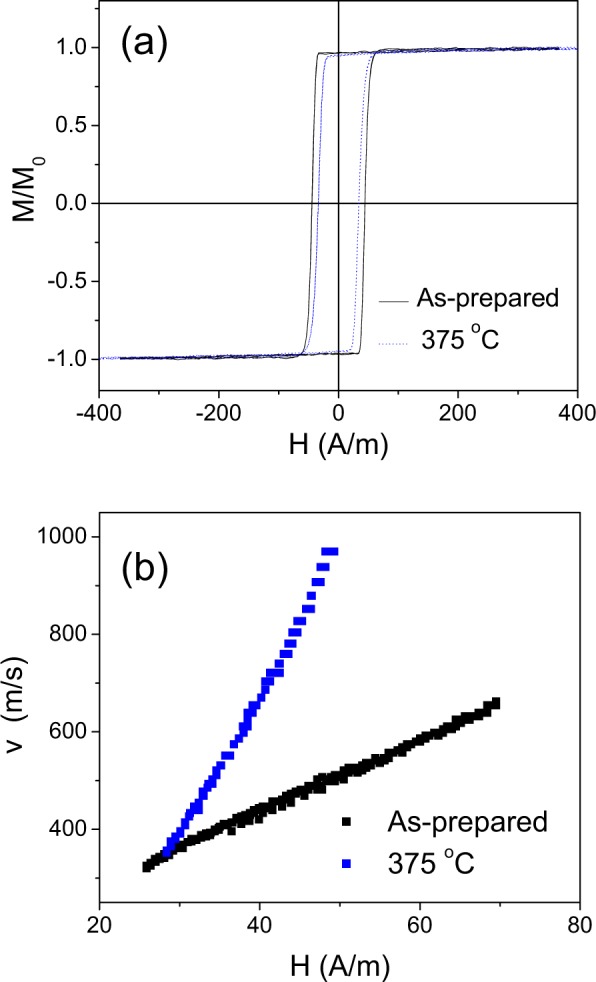
Hysteresis loops (**a**) and *v(H)* dependencies (**b**) of as-prepared and annealed at *T*_*ann*_ = 375 °C for *t*_*ann*_ = 60 min Fe_75_B_9_Si_12_C_4_ microwires.

As-prepared and heat treated (*T*_*ann*_ = 375 °C, *t*_*ann*_ = 60 min) samples, measured at room temperature, present rectangular hysteresis loops as typically observed in microwires with positive *λ*_*s*_ –values (Fig. 1a)^4,31^. After annealing (i.e., *T*_*ann*_ = 375 °C, see Fig. 1a) the hysteresis loop remains its rectangular shape, but some coercivity, *H*_*c*_, decreasing is observed.

Accordingly, single DW propagation is checked both in as-prepared and in annealed Fe_75_B_9_Si_12_C_4_ microwires (see Fig. 1b) measured at room temperature.

The DW mobility, *S*, can be experimentally evaluated from the linear dependence of the DW velocity, *v*, versus the applied magnetic field, *H*, in a viscous regime using the expression^4,36,47^:

2 $$\nu =S(H-{H}_{0})$$

where *H*_0_ is the critical propagation field, below which the domain wall propagation is not possible.

**Figure 2:**
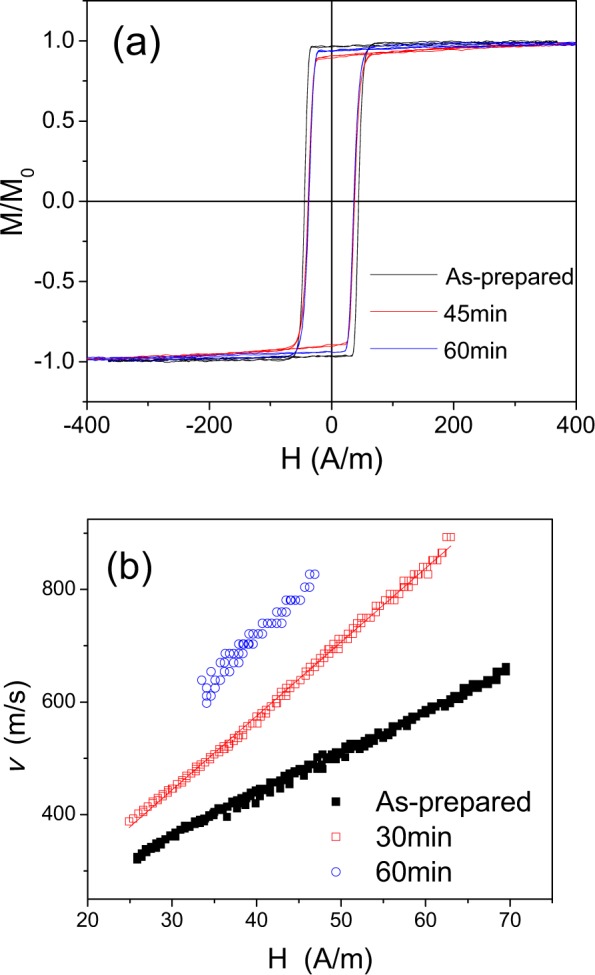
Hysteresis loops (**a**) and *v(H)* dependencies (**b**) of as-prepared and annealed at *T*_*ann*_ = 325 °C Fe_75_B_9_Si_12_C_4_ microwire.

A noticeable increase of DW velocity, *v*, and DW mobility, *S*, are observed after annealing (see Fig. 1b). Similarly increase of *S* and *v*-values is observed increasing the annealing time, *t*_*ann*_ (see Fig. 2).

As can be observed, DW dynamics of as-prepared and annealed Fe-rich microwires present practically perfectly linear *v(H)* tendencies (Figs 1b and 2b).

Additionally, *v* and *S*-values are affected by *T*_*ann*_ (*v* and *S*-values observed for *T*_*ann*_ = 375 °C are higher than those for *T*_*ann*_ = 325 °C, see Figs 1b and 2b).

Similar DW velocity increasing in annealed at different temperatures Fe- and Fe-Ni -rich microwires was recently reported^31,33,48^.

As mentioned elsewhere^26–28^, the majority of internal stresses are related to the difference between the thermal expansion coefficients of the metallic nucleus and glass coating.

Previously the dependence of *v* and *S*- values on annealing conditions has been discussed considering the relation of the domain wall mobility, *S*, and the domain wall width, *δ*_*w*_^31,33^:

3 $$S \sim {\delta }_{w} \sim {(A/K)}^{1/2}$$

where *A* is the exchange stiffness constant and *K* is the magnetic anisotropy constant.

Indeed, in the absence of the magnetocrystalline anisotropy the magnetoelastic anisotropy given by Eq. (1) becomes one of the main sources of magnetic anisotropy of amorphous glass-coated microwires.

Accordingly, increase of *S* and *v*-values can be related to the stress relaxation.

Recently we have reported that stress annealing of Fe-rich microwires results in induced transverse magnetic anisotropy that can be beneficial for further DW velocity improvement^36,37,43,44^. Therefore, we performed stress annealing of the Fe_75_B_9_Si_12_C_4_ microwire.

**Figure 3:**
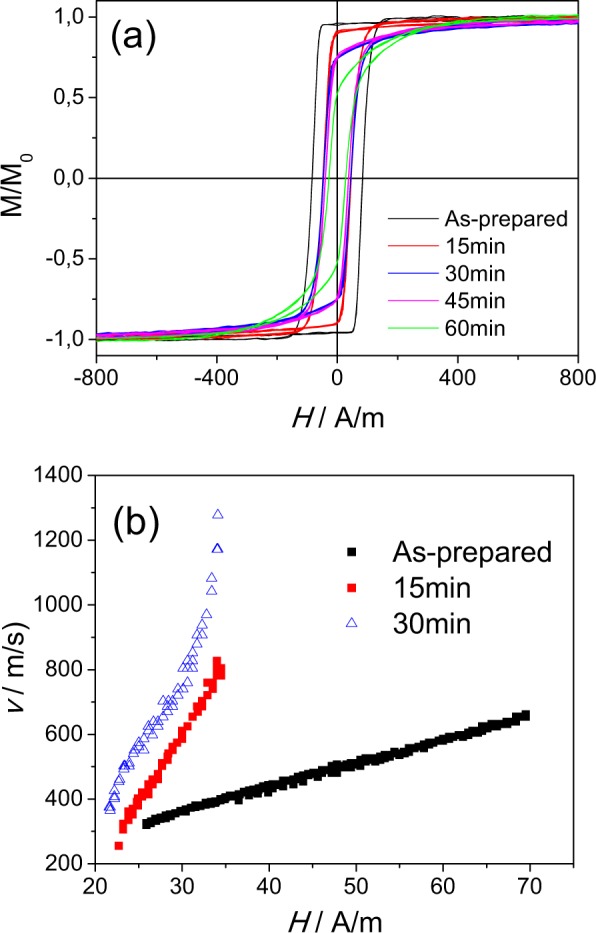
Hysteresis loops (**a**) and *v(H)* dependencies (**b**) of as-prepared and stress- annealed at *T*_*ann*_ = 325 °C and *σ*_*m*_ = 190 MPa Fe_75_B_9_Si_12_C_4_ microwires.

As can be appreciated from Fig. 3, measured at room temperature, noticeable magnetic softening of studied microwire and even disappearance of rectangular hysteresis loops is observed with stress-annealing time, *t*_*ann*_, increasing. However, up to *t*_*ann*_ = 30 min the hysteresis loop maintain the rectangular character. Evaluated *v(H)* dependencies present drastic increase in DW velocity and mobility in stress –annealed samples.

The mobility values, *S*, calculated from Figs 2 and 3 for as-prepared Fe_75_B_9_Si_12_C_4_ sample gives about 7 m^2^/As, and after been annealed at 325 °C *S*-values increase drastically up to *S* ≈ 10 m^2^/As.

Upon the stress- annealing time the DW mobility remarkably increases achieving values almost about *S* ≈ 40 m^2^/A∙s (see Fig. 3b). The range of linear approximation for *S*-value, for the sample stress- annealed at 325 °C during 30 minutes, was from 22 up to 34 A/m, where the *v(H)* dependence maintains linear. For H > 34 A/m the deviation from *v(H)* dependence is observed. The reasons for such deviation will be discussed later.

**Figure 4:**
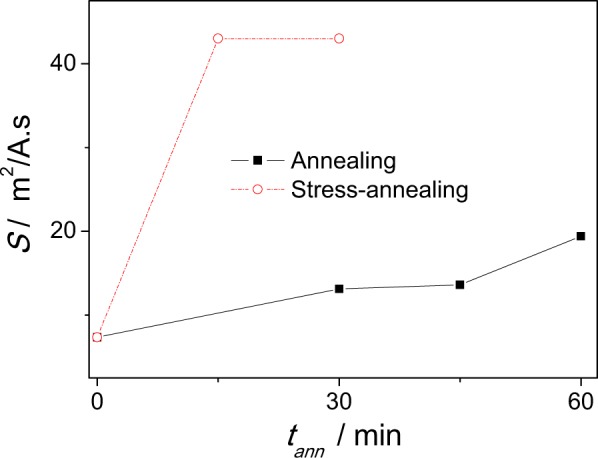
DW mobility, *S*, as a function of annealing time for Fe_75_B_9_Si_12_C_4_ microwires annealed at *T*_*ann*_ = 325 °C.

Comparison of *S*-values evaluated in the sample subjected to conventional annealing and stress-annealing at 325 °C is shown in Fig. 4.

The domain wall, *S*, is the domain wall mobility given by^49,50^:

4 $$S=2{\mu }_{0}{M}_{s}/\beta $$

where *β* is the viscous damping coefficient, μ_0_ is magnetic permeability of vacuum.

Various contributions to the domain wall damping have been analyzed elsewhere^26,31,32,50^.

Generally three contributions have been considered^50^:

The eddy current contribution, *β*_*e*_, is associated to the micro-eddy currents circulating nearby moving domain wall. Generally, the eddy current parameter, *β*_*e*_, is considered to be negligible in high-resistive amorphous materials^26,31^ like thin amorphous microwires. However, the analysis eddy current contribution for a planar domain wall moving in a cylindrical wire with an outer shell of radial domains shows that *β*_*e*_ depends on radius of the metallic nucleus and the axially magnetized domain^50^. Therefore, later we will analyze the evolution of the domain structure upon stress-annealing.

The magnetic relaxation damping, *β*_*r*_, is the second contribution. This damping is related to the Gilbert damping parameter, *α*, and is inversely proportional to the domain wall width *δw*^50^,

5 $${\beta }_{r}\approx 2\alpha {\pi }^{-1}{(K/A)}^{1/2}$$

where *A* is the exchange interaction constant, *K* is the coefficient of uniaxial anisotropy, and *α*-is the so-called Gilbert damping coefficient. As mentioned above, in amorphous materials the main origin of magnetic anisotropy is the magnetoelastic anisotropy, *K*_*me*_, given by (1).

However, as reported^51^ *α* in amorphous microwires does not depend on annealing. Therefore, internal stresses relaxation can affect *β*_*r*_ through *K*_*me*_.

The third contribution is the structural relaxation contribution originated from the interaction of mobile defects with the local magnetization^50^. Generally, this contribution is usually neglected in the viscous regime.

The other particularity of amorphous alloys is the possibility of the DW stabilization that usually is manifested as considerable coercivity growth upon annealing at temperatures below Curie temperature^52,53^. However, in the present case we did not observe any coercivity increasing. Instead, coercivity decreasing can be appreciated from Figs 1–3. Therefore, the origin of drastic increasing of *v* and *S*- values upon stress annealing (as-compared to annealing) must be related to the influence of the transverse magnetic anisotropy related to the stress-annealing.

**Figure 5:**
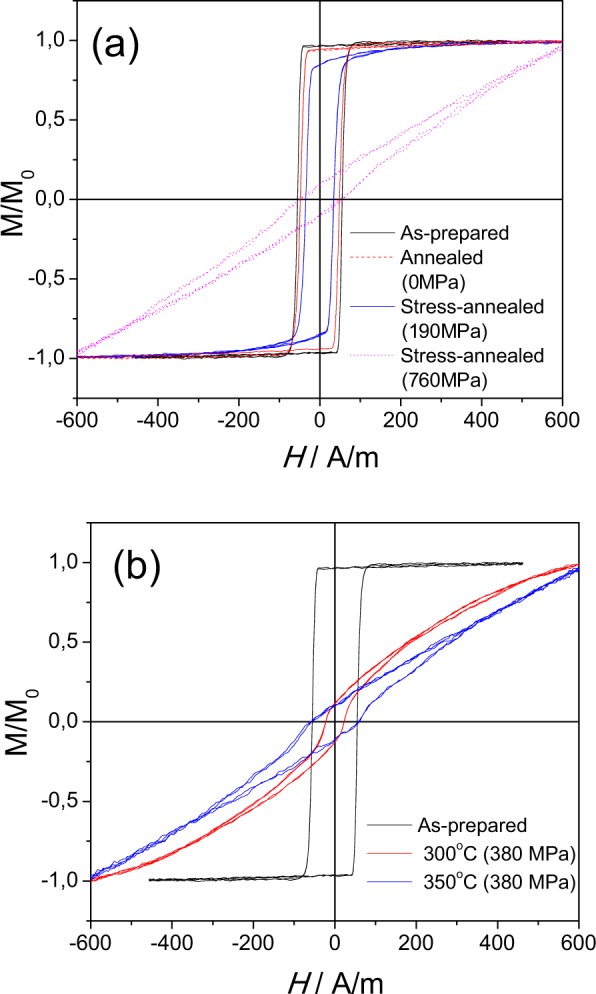
Hysteresis loops of studied samples annealed at *T*_*ann*_ = 300 °C for *t*_*ann*_ = 60 min (**a**) and annealed at *σ*_*appl*_ = 380 MPa for *t*_*ann*_ = 30 min at different temperatures (**b**).

Observed stress-induced anisotropy is considerably affected not only by annealing time (as evidenced from Fig. 3a), but also by various factors, like annealing temperature, *T*_*ann*_, or stress applied during the annealing, *σ*_*appl*_, as can be appreciated from Fig. 5a,b. At certain stress annealing conditions, i.e., high enough *T*_*ann*_ and *σ*_*appl*_, the hysteresis loop is no more rectangular: change from rectangular hysteresis loop to almost linear is observed (Fig. 5).

Considering that the magnetic domain structure of magnetic wires is assumed to be consisting of outer domain shell with transverse magnetization orientation and inner axially magnetized core^8,30,54^, one can evaluate the domain structure modification from the squareness ratio, *M*_*r*_*/M*_*s*_ as^54^:

6 $${R}_{c}=R{({M}_{r}/{M}_{s})}^{l/2},$$

where *R* is the metallic nucleus radius.

**Figure 6:**
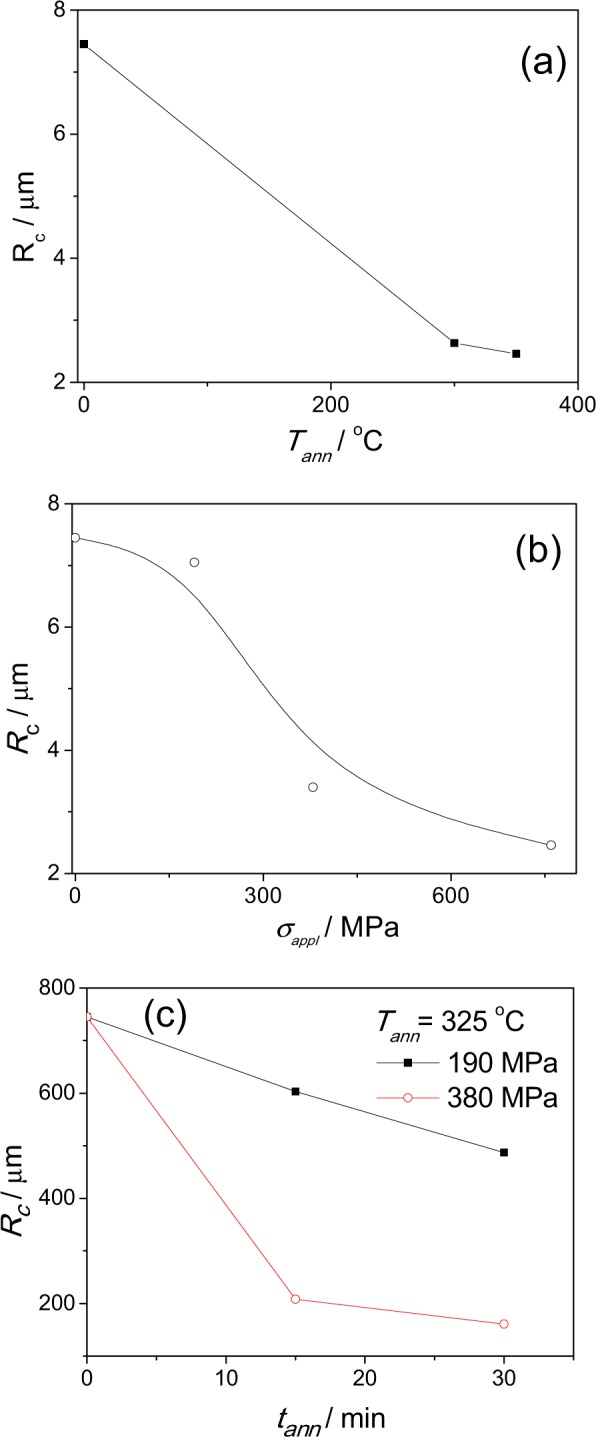
Effect of annealing temperature (**a**), stress applied during annealing at *T*_*ann*_ = 300 °C (**b**) and annealing time (**c**) on *R*_*c*_-values of studied microwire.

In this way from *M*_*r*_*/M*_*s*_-values obtained from hysteresis loops presented in Fig. 5 we evaluated the dependence of the radius of inner axially magnetized core, *R*_*c*_, on annealing conditions. As can be appreciated from Fig. 6, *R*_*c*_ -values progressively decrease with increasing of *σ*_*appl*_, *T*_*ann*_ and *t*_*ann*_-values.

At fixed annealing temperature the radius of inner axially magnetized core, *R*_*c*_, is lower at higher applied stress (Fig. 6b,c).

From aforementioned analysis we can deduce that the stress-annealing allows the increase of the volume of outer domain shell with transverse magnetization orientation increase in expense of decreasing of the radius of inner axially magnetized core.

Consequently, beneficial effect of transverse magnetic anisotropy on DW velocity (see Figs 1–4) must be attributed to the increase of the volume of outer domain shell with transverse magnetic anisotropy.

We can assume that this outer domain shell with transverse magnetic anisotropy affects the travelling DW in a similar way as application of transversal bias magnetic field allowing enhancement the DW velocity^36,37,42,43^.

Recently we reported that stress-induced transversal magnetic anisotropy in Fe-rich microwires can be beneficial for the magnetoimpedance effect optimization^44–46^.

As consequence, one can expect improvement of the GMI effect by stress-annealing in the present case as well.

**Figure 7:**
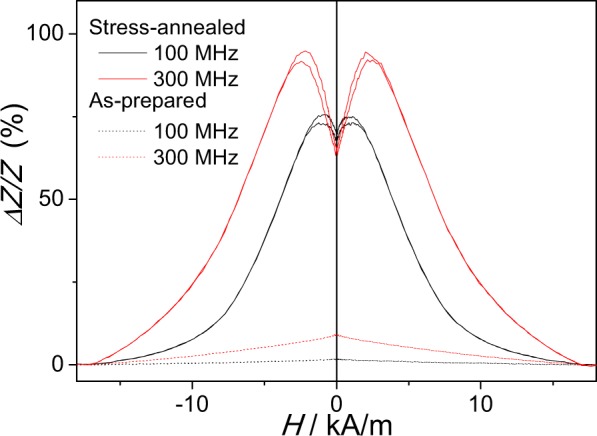
*ΔZ/Z(H)* dependencies of as-prepared and stress-annealed at 350 °C (*σ*_*appl*_ = 380 MPa) for *f* = 100 and 300 MHz.

Indeed, from experimental results provided in Fig. 7 we can observe remarkable increasing of the GMI ratio. Comparison of maximum *ΔZ/Z* values of as-prepared and stress-annealed samples evidences an order of magnitude increasing of *ΔZ/Z*-values (from about 10% at 300 MHz to about 100%). Additionally, not only the *ΔZ/Z*-value, but also the Δ*Z/Z(H)* dependence for as-prepared and stress-annealed samples present considerable difference. As-prepared Fe_75_B_9_Si_12_C_4_ microwires exhibit the typical behaviour of magnetic microwires with axial magnetic anisotropy, i.e., decay with magnetic field increasing (see Fig. 7).

Stress-annealed microwires present double-peak *ΔZ/Z(H)* dependence previously associated with magnetic wires with circumferential magnetic anisotropy^55^.

As previously discussed elsewhere^56^, one of the obstacles limiting applications of fast DW propagation observed in micro- and nano-wires is that the travelling DW is essentially not abrupt. However, it was previously reported^39^, that the characteristic width *δ* of a head-to-head DW is closely related to the magnetoelastic anisotropy. Thus, the reduced head-to-head domain wall width *δ/d* (*d* is the metallic nucleus diameter) is determined by the value of the anisotropy constant *K*: for *K* = 10^4^ erg/cm^3^ *δ/d* ≈ 13.5 and for *K* = 10^3^ erg/cm^3^ *δ/d* = 40–50^39^. For these estimations it was assumed that the whole volume of the metallic nucleus diameter presents axial magnetization.

In the present case we are able to tune the volume of the inner axially magnetized core by annealing time and stress applied during the annealing (see Fig. 6). Therefore, we may expect the modification of DW characteristic width *δ* upon stress annealing.

The characteristic DW width can be evaluated from the EMF signals generated by a head-to-head DW moving through the microwire^39,40^.

As described elsewhere^39,40^, the EMF generated within the turn of the pick-up coil by a change in the magnetic flux can be expressed as:

7 $$\varepsilon (t)=\frac{{\rm{\Delta }}\varphi }{{\rm{\Delta }}t}$$

where *Φ* = *BS* is the magnetic flux, *S* is the area of the surface, *B* = *M* + *H* is the magnetic induction, and *M* is the magnetization. Thus, the features (the amplitude and width) of the EMF peaks must be determined by $\frac{\partial M}{\partial t}$.

**Figure 8:**
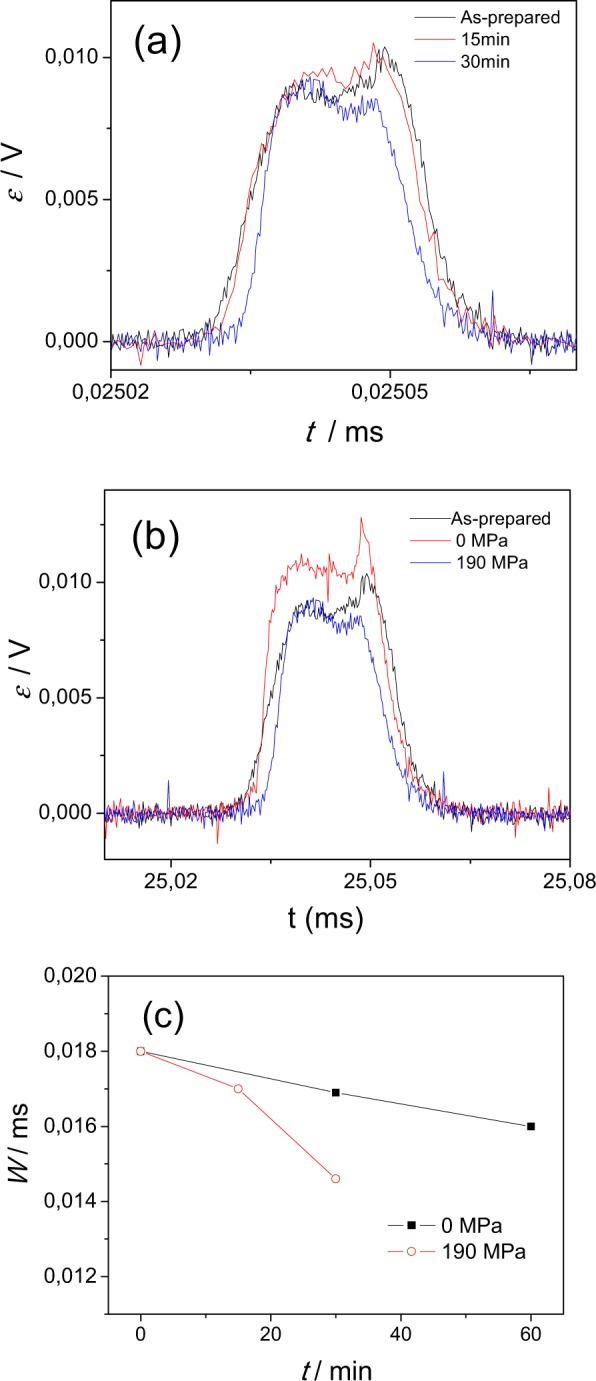
EMF peaks induced by magnetization changes in pick-up coil measured for (**a**) as-prepared and stress-annealed (at *σ*_*appl*_ = 190 MPa) microwires for different annealing times, (**b**) in both as-prepared and annealed at 325 °C under stress and without stress and (**c**) evolution of the half-width of the EMF signal after annealing. The estimations are made for H = 25 A/m.

As can be appreciated from Fig. 8, a decreasing of the EMF signal width from the pick-up coil can be appreciated after stress-annealing. The EMF signals, *ε*, have been compared for as-prepared and stress-annealed at different times samples (Fig. 8a), as well as for as-prepared and those annealed under stress and without stress (Fig. 8b).

Such changes are evidenced by the evaluation of the half-width, *W*, of the EMF signal with annealing time provided in Fig. 8c. As can be appreciated, a decrease of half-width of the EMF signal after stress-annealing is evidenced.

As discussed above, such decreasing of the half-width (full width at half maximum), *W*, must be associated either to the decreasing of the characteristic DW width or to the DW velocity increasing. The reason for such modifications can be stress-annealing induced transverse magnetic anisotropy as well as reduction of the volume of the inner axially magnetized core after stress-annealing. Indeed as mentioned above, the *δ* –values are determined by the magnetoelastic anisotropy and by the diameter of the axially magnetized core.

In order to separate these two factors we must analyze in more detail the EMF generated within the pick-up coil. Previously the EMF, *ε*, generated within the pick-up coil turn when DW width, *δ*, is comparable with the distance to the coil turn, *z*, was analyzed^39^. The expression obtained in this case is^39^:

8 $$\varepsilon (t)=-\,QV{R}^{2}\frac{\sqrt{\pi }}{2}\int d{z}_{1}\frac{\langle \frac{\partial {\alpha }_{z}}{\partial {z}_{1}\,}({z}_{1}-Vt)\rangle }{{({(z-{z}_{1})}^{2}+{R}^{2})}^{3/2}}$$

where *R* is the radius of the coil turn, *V* = *−dz/dt*- domain wall velocity, $\frac{\partial {\alpha }_{z}}{\partial {z}_{1}}$ is the linear density of the DW magnetic charge averaged over the wire cross section, Q- magnetic charge.

The Eq. (8) is rather complex. In the simplified case, when the characteristic domain wall width, *δ*, is small compared with the distance z from the coil turn to the DW position, the Eq. (8) can be simplified as^39^:

9 $$\varepsilon (t)=-\,\frac{\sqrt{\pi }}{2}\frac{QV{R}^{2}}{{(z+{R}^{2})}^{3/2}}$$

We can compare the EMF signals for as-prepared and stress-annealed samples if we consider the same coil parameters.

In this case the only difference in EMF values must be associated to the different DW velocity, *v*, values and difference in remanent magnetization of as-prepared and stress- annealed samples. The latter contributes through the magnetic charge, *Q* = *2Mr S*, where *S* is the sample cross section and *M*_*r*_ – remanent magnetization and is attributed to the fact that only the remagnetization reversal of the inner axially magnetized core contributes to the EMF signal.

These considerations allows us to evaluate if the difference in half-width of the EMF signal of as-prepared and stress- annealed (*T*_*ann*_ = 325 °C *σ*_*appl*_ = 190 MPa, *t*_*ann*_ = 30 min) microwires is attributed only to different DW velocities or if DW shape change after stress annealing takes place too. Obtained velocities ratio taken from Figs 2 and 3 for *H* = 25 A/m for stress annealed and as-prepared samples (*v*_*sa*_ and *v*_*ap*_, respectively) gives *v*_*sa*_*/v*_*ap*_ ≈ 1.25. However, considering the difference in the remanent magnetization (evaluated from Figs 2a and 3a), the ratio *Q*_*sa*_ *v*_*sa*_/*Q*_*ap*_ *v*_*ap*_ *≈* 0.98 (where *Q*_*sa*_ and *Q*_*ap*_ are values for stress-annealed and as-prepared samples). While the *W* –values ratio, i.e. *W*_*sa*_/*W*_*ap*_ (where *W*_*ap*_ and *W*_*sa*_ are the half-width of the EMF peaks for as-prepared and stress-annealed samples) is about 0.83.

**Figure 9:**
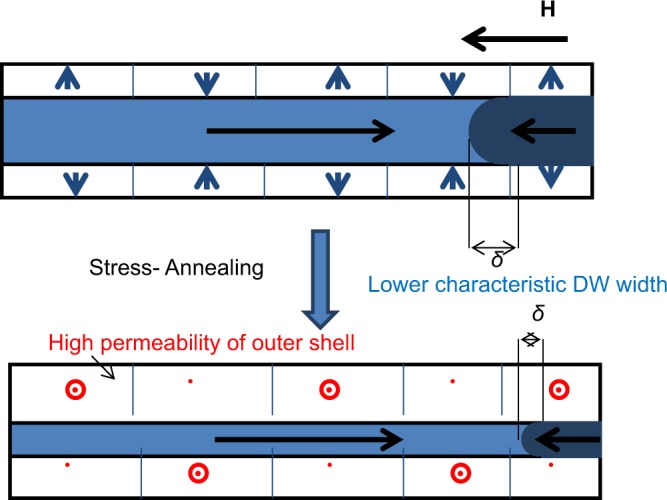
Schematic picture illustrating the influence of stress-annealing induced anisotropy on magnetic properties of microwires.

Consequently, we can assume that the characteristic DW width reduction in stress-annealed microwires takes place as schematically shown in Fig. 9.

On the other hand, a remarkable DW velocity and mobility improvement appreciated in stress-annealed samples must be related to stress-annealing induced transverse magnetic anisotropy.

A significant increase of the DW velocity as well as DW width for a given driving field below the Walker breakdown field is recently reported for nanowires upon application of transverse magnetic field or by varying the width and thickness of planar nanowires^42,43,57,58^. Such effect of transverse magnetic field on DW dynamics is discussed considering the influence of magnetic field on structure of DW, on the DW energy landscape which affects the DW features and Walker breakdown field. In the case of tunning the DW dynamics by varying the width and thickness of planar nanowires the magnetic anisotropy field has been tuned by the shape anisotropy^58^. In the present case we observed similar effect when the transverse magnetic anisotropy is induced by stress annealing.

Considering aforementioned evolution of domain structure upon annealing (increase of the volume of the outer domain shell with transverse magnetic anisotropy at the expense of the inner axially magnetized core) we can assume that arising of stress-annealing induced transverse magnetic anisotropy is somehow equivalent to the microwire diameter reduction.

As mentioned above the deviation from linear *v(H)* dependence is observed for stress-annealed microwire (T_ann_ = 325 °C, *t*_*ann*_ = 30 min). One possible explanation for the slope change could be attributed to the change of the domain wall structure. Indeed, as follows from Fig. 6c, the inner core radius decrease upon stress annealing can be deduced.

As discussed elsewhere, the length of DW depends on applied field^39,41^. However, if the DW changes its shape, one can expect changes in the stray field (due to the new wall shape) and hence variation of DW mobility^41^. In the present case of stress-annealed sample with thicker outer domain shell we can consider that upon applied magnetic field the remanence will increase. Such increase of remanence must be associated to rising of the inner axially magnetized core volume and hence in DW shape change: generally higher DW mobility is expected for longer DW^41,59^. Consequently, one can expect non-linear *v(H)* dependence for stress-annealed sample with lower remanent magnetization. It is worth mentioning that such behaviour is predicted for planar DWs, however the DW shape in microwires is quite complex: long planar, and conical DWs are proposed for magnetic microwires^59–61^.

Recently, the origin of stress-annealing induced transverse magnetic anisotropy in Fe-rich microwires is discussed in terms of either “back stresses” leading to the redistribution of the internal stresses or topological short range ordering^44–46,52^.

Indeed, as reported elsewhere^52,62–65^, annealing of amorphous magnetic materials under stresses and/or magnetic field can affect their macroscopic magnetic anisotropy. Such induced magnetic anisotropy of amorphous materials has been attributed to either compositional^52^ or topological short- range ordering^62^. The compositional short- range ordering is usually associated with directional ordering of atomic pairs^52^. However, the origin of the topological short range ordering is attributed to either angular distribution of the atomic bonds^62^ or anisotropic structural rearrangements in vicinity of the glass transition temperature^65^.

Aforementioned directional ordering of atomic pairs explains well features of induced magnetic anisotropy in amorphous alloys with two or more magnetic elements^62–65^. Therefore, for the present case of Fe_75_B_9_Si_12_C_4_ amorphous microwires the directional ordering of atomic pairs mechanism of observed induced magnetic anisotropy can be disregarded.

In the present case of glass-coated microwires the presence of the glass-coating induces strong internal stresses^27,28^. Therefore, previously we explained observed stress-annealing induced transverse magnetic anisotropy considering the so-called “back stresses” arising from glass-coating and compensating internal stresses induced during fabrication process with the axial component predominant in most of the metallic nucleus volume^44–46^.

The compensation of the internal stresses with predominant axial tensile character within the main part of the metallic nucleus by compressive “back stresses” can be associated to diminishing of the magnetoelastic anisotropy and hence in rising of the DW velocity and mobility.

It is worth mention that the topological short- range ordering can provide similar stress and annealing temperature dependence and therefore can be considered.

It is worth mentioning that the devitrification of Fe-rich microwires can also provide magnetic softening and GMI effect enhancement^11,12^. However, the advantage of proposed approach involving stress annealing is that amorphous materials hold superior mechanical properties, such as plasticity and flexibility.

## Material and Methods

Amorphous Fe_75_B_9_Si_12_C_4_ (*d* = 15,2 μm; *D* = 17,2 μm) glass-coated microwires with positive magnetostriction coefficient have been prepared by the Taylor-Ulitovsky method previously described elsewhere^4,31,32^.

Samples have been annealed (without applied stress and under stress) at annealing temperatures, *T*_*ann*_, between 250 and 375 °C and annealing time, *t*_*ann*_, between 15 and 90 min in a conventional furnace.

The mechanical load (that produced the tensile stress) has been attached to the sample during the annealing and slow sample cooling with the furnace.

The stress value within the metallic nucleus and glass shell during the heat treatment has been evaluated as described earlier^45^:

10 $${\sigma }_{m}=\frac{K\cdot P}{K\,{S}_{m}+{S}_{gl}},\,{\sigma }_{gl}=\frac{P}{\,K\,{S}_{m}+{S}_{gl}}$$

where *P* is the applied mechanical load, *K* = *E*_2_/*E*_1_, *E*_*i*_ are the Young's moduli at room temperature of the metallic alloy (*E*_2_) and the glass (*E*_1_), and *S*_*m*_ and *S*_*gl*_ are the metallic nucleus and glass coating cross sections respectively. We varied the applied tensile stress, *σ*_*m*_, from 190 to 760 MPa.

Amorphous structure has been checked by X-ray Diffraction (XRD) employing a BRUKER (D8 Advance) X-ray diffractometer with Cu K_*α*_ (*λ* = 1.54 Å) radiation. All XRD patterns of the as-prepared and heat treated samples presented broad halo typical for amorphous materials. The samples crystallization was also evaluated through the differential scanning calorimetry (DSC) measurements performed at a heating rate of 10 K/min using the DSC 204 F1 Netzsch calorimeter. Beginning of crystallization of studied microwire is observed at about 522 °C.

Hysteresis loops have been measured using fluxmetric method previously described elsewhere^29^. Bulk hysteresis loops were measured with the sample placed in a pick-up coil connected to a fluxmeter, the magnetic field was generated by a solenoid and the hysteresis loops were obtained by a computer connected to the fluxmeter and the power source. We represent the normalized magnetization, *M/M*_0_ versus magnetic field, *H*, where *M* is the magnetic moment at given magnetic field and *M*_0_ is the magnetic moment of the sample at the maximum magnetic field amplitude, *H*_*m*_.

The DW velocity has been evaluated using modified Sixtus-Tonks technique^4,31–34,37,38^. The main difference of used method with respect to classical Sixtus-Tonks method^9^, is that one wires end is placed outside the magnetization coil to ensure a single DW propagation from the opposite wire end. Additionally, in order to avoid exaggerated DW velocity values related to the multiple DW propagation we employed 3 pick-up coils^4,37,38^. Using this method we can estimate the DW velocity as:

11 $$\nu =\frac{1}{{\rm{\Delta }}t}$$

where *Δt* is the time difference between the induced electromotive force (EMF) peaks and *l* is the distance between a pair of pick-up coils. Consequently, we can evaluate the DW velocity between the first and the second, the second and the third and the first and the third pick-up coils, *v*_*1–2*_, *v*_*2–3*_ and *v*_*1–3*_ respectively^4,37,38^.

We considered only the linear region of *v(H)* corresponding to viscous DW propagation disregarding the deviations from linear *v(H)* dependencies at high field region. Previously such deviations have been discussed considering either Walker regime^32,34^, or a multiple DW nucleation at defects^4,37,38^.

For the GMI effect characterization we used a micro-strip sample holder placed inside a long solenoid producing a magnetic field, *H*^44–46^. The impedance, *Z*, has been evaluated from the reflection coefficient, *S*_11_, measured using the vector network analyzer as:

12 $$Z={Z}_{0}(1+{S}_{11})/(1-{S}_{11}),$$

where *Z*_0_ = 50 Ohm is the characteristic impedance of the coaxial line.

The GMI ratio, *ΔZ/Z*, is defined as:

13 $${\Delta }Z/Z=[Z(H)-Z({H}_{max})]/Z({H}_{max}),$$

where *H*_*max*_ is the maximum applied DC magnetic field.

## Conclusions

The impact of stress-annealing on magnetic properties and domain wall (DW) propagation and giant magneto-impedance (GMI) effect in Fe_75_B_9_Si_12_C_4_microwires is experimentally studied. Observed stress-induced anisotropy is considerably affected by annealing conditions (annealing time, temperature or stress applied during the annealing).

Remarkable improvement of DW mobility (from 7 m^2^/A∙s to 40 m^2^/A∙s) and GMI ratio (from about 10% at 300 MHz to about 100%) are achieved by stress-annealing. Additionally, not only the *ΔZ/Z*-value, but also Δ*Z/Z(H)* dependencies for as-prepared and stress-annealed samples present considerable difference. A remarkable influence of stress-annealing on hysteresis loops is associated to the domain structure modification. From the hysteresis loops we evaluated the dependence of the radius of inner axially magnetized core on annealing conditions.

Beneficial effect of stress-annealing on GMI effect and DW dynamics is attributed to the induced transverse magnetic anisotropy. An improvement of the circumferential permeability in the metallic nucleus surface layer is evidenced from observed magnetic softening and growing volume of outer domain shell with transverse magnetic anisotropy. We assumed that this outer domain shell with transverse magnetic anisotropy affects the travelling DW in a similar way as application of transversal bias magnetic field allowing enhancement the DW velocity. Decreasing of the half-width of the EMF peak in stress-annealed microwires has been associated to the decrease in the characteristic DW width.

Accordingly, stress annealing enabled us to achieve the magnetic anisotropy distribution beneficial for optimization of either the GMI effect or the DW dynamics.

Observed dependencies are discussed taking into account the effects of magnetoelastic anisotropy, internal stresses relaxation after annealing and stress-induced magnetic anisotropy.

Consequently, stress-annealing is the effective method for engineering of the DW dynamics, magnetic softness and GMI effect in Fe-rich glass-coated microwires.
